# Finite Element Modelling Simulated Meniscus Translocation and Deformation during Locomotion of the Equine Stifle

**DOI:** 10.3390/ani9080502

**Published:** 2019-07-31

**Authors:** Pasquale Zellmann, Iris Ribitsch, Stephan Handschuh, Christian Peham

**Affiliations:** 1 Department for Companion Animals and Horses, University Equine Hospital, Vetmeduni Vienna, 1210 Vienna, Austria; 2 VetCore Facility for Research, Imaging Unit, Vetmeduni Vienna, 1210 Vienna, Austria

**Keywords:** horse, stifle, meniscus, finite element analysis, finite element model

## Abstract

**Simple Summary:**

Meniscal tears are one of the most common soft tissue injuries in the equine stifle joint. To date no optimal treatment strategy to heal meniscal tissue is available. Accordingly, there is a need to improve treatment for meniscal injuries and thus to identify appropriate translational animal models. A possible alternative to animal experimentation is the use of finite element modelling (FEMg). FEMg allows simulation of time dependent changes in tissues resulting from biomechanical strains. We developed a finite element model (FEM) of the equine stifle joint to identify pressure peaks and simulate translocation and deformation of the menisci at different joint angles under loading conditions. The FEM model was tested across a range of motion of approximately 30°. Pressure load was higher overall in the lateral meniscus than in the medial meniscus. Accordingly, the simulation showed higher translocation and deformation throughout the whole range of motion in the lateral compared to the medial meniscus. The results encourage further refinement of this FEM model for studying loading patterns on menisci and articular cartilages as well as the resulting mechanical stress in the subchondral bone. A functional FEM model can not only help identify segments in the femoro–tibial joint which are predisposed to injury, but also provide better understanding of the progression of certain stifle disorders, simulate treatment/surgery effects and to optimize implant/transplant properties in order to most closely resemble natural tissue.

**Abstract:**

We developed a finite element model (FEM) of the equine stifle joint to identify pressure peaks and simulate translocation and deformation of the menisci. A series of sectional magnetic resonance images (1.5 T) of the stifle joint of a 23 year old Shetland pony gelding served as basis for image segmentation. Based on the 3D polygon models of femur, tibia, articular cartilages, menisci, collateral ligaments and the meniscotibial ligaments, an FEM model was generated. Tissue material properties were assigned based on data from human (Open knee(s) project) and bovine femoro-tibial joint available in the literature. The FEM model was tested across a range of motion of approximately 30°. Pressure load was overall higher in the lateral meniscus than in the medial. Accordingly, the simulation showed higher translocation and deformation in the lateral compared to the medial meniscus. The results encourage further refinement of this model for studying loading patterns on menisci and articular cartilages as well as the resulting mechanical stress in the subchondral bone (femur and tibia). A functional FEM model can not only help identify segments in the stifle which are predisposed to injury, but also to better understand the progression of certain stifle disorders, simulate treatment/surgery effects and to optimize implant/transplant properties.

## 1. Introduction

The femoro–tibial joint is one of the biggest and most complex mammalian joints. Based on its six degrees of freedom (three translational and three rotational) it facilitates not only flexion and extension but also slight internal and external rotation [1,2,3]. However, it is prone to injury and consequently the development of secondary osteoarthritis (OA)—one of the leading causes of disability worldwide [4].

Commonly injured structures inside the femoro–tibial joint are the menisci, which provide stability and congruency to the otherwise incongruent joint, contribute to lubrication, reduce tibiofemoral contact pressure and dissipate shock [5]. However, only intact menisci are able to properly fulfil these biomechanical functions and OA is therefore often incurred in consequence of meniscal tears [6].

Accordingly, the need to develop sustainable therapies for the treatment of meniscus injuries is high. An integral part of developing new therapies is animal testing. Animal models offer a close approximation of the disease pathophysiology and cannot yet be completely replaced. However, the number of experiments involving live animals should be reduced to a minimum [7]. A possible alternative to animal experimentation is the use of finite element analysis (FEA). FEA has been used in Orthopaedics since 1972 [8] and allows us to simulate time dependent changes in tissues resulting from biomechanical wear and tear [9]. Hence FEA modelling is hoped to contribute to a better understanding of disease and injury mechanisms, which is the basis for a better diagnosis, treatment and development of an effective prevention program [10,11].

An important requirement for any suitable model, hence also for FEA models, is closest possible analogy to the human. The gait of horses is considered to resemble the humans [12] with a range of motion of 150° [2] from full flexion to full extension. Other analogies which predestine the horse as an animal model for human femoro–tibial joint disorders in general and meniscus disorders in particular are the similar histology [13] as well as craniocaudal translocation of equine and human menisci during femoro–tibial joint flexion and extension [14]. Furthermore, horses suffer from naturally occurring meniscus injuries [1,15,16,17,18] which are reported to be one of the most common soft tissue injuries in the equine stifle joint (68% of all soft tissue stifle disorders) [19]. Similar to humans, concomitant articular cartilage damage leading to secondary OA has been reported in 71% of equine meniscus injury cases [20]. Hyperextension of the stifle can cause pathological forces and injuries in the cranial horn of the equine medial meniscus, analogous to those observed in the human medial posterior meniscal horn upon hyperflexion [21,22]. The structural as well as functional analogies and the shared risk of meniscus injures make the horse not only a valid animal model, but also a potential beneficiary of advanced knowledge in meniscus biomechanics.

While there are lots of different finite element models (FEM) of the human femoro–tibial joint [23,24,25,26,27], currently no FEM is available for the equine stifle. A FEM could help unravel equine stifle joint mechanics and identify pathologic loads or load distribution patterns as possible causes of injury and consequent degeneration. This would yield clear benefits for equine patients and further substantiate the horse as a valid animal model for human knee disorders. In the current paper the FEA was used to explore the extent of meniscal extrusion and contact pressure distribution and to simulate meniscus’ translocation and deformation during locomotion. The model was checked for plausibility based on data available in the literature [1,2].

To our knowledge this is the first prototype of an FEA model of the equine menisci simulating the effect of the joint angle to pressure distribution.

## 2. Materials and Methods

### 2.1. Study Animal and Image Acquisition

The FEM of the equine stifle joint was created based on magnetic resonance imaging (MRI) data of a formalin-fixed left hind limb of a 23 year old Shetland pony gelding (96 kg body mass). The study did not require ethical approval as it was performed in a dead horse, which died for reasons unrelated to this study. However, the animal’s owner’s consent to dissect and analyze the leg and to publish resulting data was obtained according to the standard procedure, which was approved by the ethics and animal welfare committee of the University of Veterinary Medicine Vienna.

The limb was dissected according to the “Good Scientific Practice and Ethics in Science and Research” regulation implemented at the University of Veterinary Medicine. The horse showed no signs of a current or previous orthopaedic disorder of the stifle joint. MRI was performed using a Siemens Magnetom Espree 1.5 Tesla (Siemens Healthineers, Erlangen Forchheim, Germany). A transverse T2 TSE (turbo spin echo) sequence (voxel resolution 0.52 × 0.52 × 2.60 mm) and a sagittal T2 GRE (gradient-echo) sequence (voxel resolution 1.2 × 0.59 × 0.58 mm), were used for the 3D model generation. Image data were exported in DICOM format.

### 2.2. Image Processing, Image Segmentation and Generation of Polygon Surface Models

**Figure 1:**
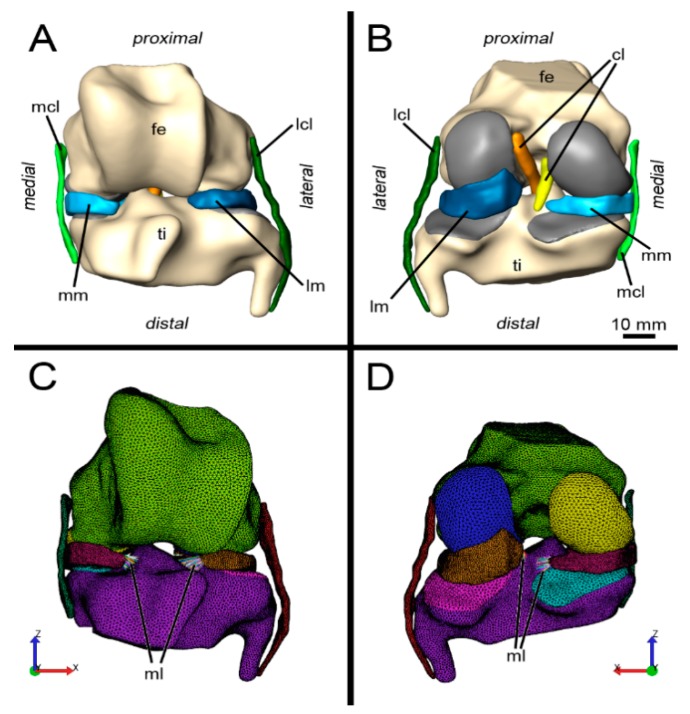
Overview of the equine stifle 3D model used for finite element analysis (FEA). (**A**) Cranial view of the 3D model of the horse stifle joint. Polygon triangle meshes were generated from image segmentation based on MRI data using the commercial 3D software package Amira 5.3. (**B**) Caudal view of the 3D model of the horse stifle joint. (**C**) Cranial view showing elements of the finite element model generated using the software package Preview of the FEBio Software Suite. Meniscal ligaments were simulated as spring tied interface contacts. (**D**) Caudal view of the elements of the finite element model.

DICOM series were imported into the 3D software package AMIRA 5.3 (Informer Technologies Inc. https://www.informer.com/). The two MRI sequences were registered using the “AffineRegistration” tool with a rigid transformation based on normalized mutual information. For both sequences, voxel resolution was virtually increased with the resample tool to 0.2 mm isotropic voxel size using the resample tool to facilitate image segmentation and to reduce artefacts related to anisotropic voxel dimensions in the original MRI data. Relevant anatomic features were manually segmented using the AMIRA “SegmentationEditor” as illustrated in the Supplementary Materials (Figure S1). The T2 GRE sequence was used for segmentation of femur, tibia, patella, menisci and collateral ligaments, while the T2 TSE sequence was used for segmentation of the cruciate ligaments and the meniscofemoral ligament. Segmentation of the articular cartilage of the femur condyles and the tibia plateau was implemented by dilation of the bone contour. Based on voxel segmentation, polygon surface models were created using the SurfaceGen tool. The surfaces were processed including a reduction in triangle number and smoothing (SmoothSurface). Surfaces were remeshed (RemeshSurface) to get homogenous triangle sizes and exported as polygon meshes consisting of triangles in STL (Standard Template Library) format (Figure 1A,B). Figure 1 shows all elements of the equine stifle 3D model as an interactive 3D PDF.

Fe = femur, cl = cruciate ligaments, lcl = lateral collateral ligament, lm = lateral meniscus, mcl = medial collateral ligament, ml = meniscal ligaments, mm = medial meniscus, ti = tibia.

Color coding for sections C and D: green = Femur; blue = cartilage surface of lateral femoral condyle; yellow = cartilage surface of medial femoral condyle; purple = Tibia; pink = cartilage surface of lateral tibia plateau; turquoise = cartilage surface of medial tibia plateau; ruby = medial meniscus; orange = lateral meniscus; dark green = medial collateral ligament; brown = lateral collateral ligament; multi colored = meniscal ligaments.

### 2.3. Computer Soft- and Hardware for Finite Element Analysis (FEA)

All other image processing including the modelling of the final FEM was performed using the FEBio software suite (University of Utah, Musculoskeletal Research Laboratories and Columbia’s Musculoskeletal Biomechanics Laboratory, Columbia, Utah, USA). The freeware selected has been validated for this purpose by previous studies [28,29,30,31,32].

The suite consists of three different software packages: Preview (Version 1.18.0, University of Utah, Musculoskeletal Research Laboratories and Columbia’s Musculoskeletal Biomechanics Laboratory, Columbia, Utah, USA), FEBio (Version 2.3.1, University of Utah, Musculoskeletal Research Laboratories and Columbia’s Musculoskeletal Biomechanics Laboratory, Columbia, Utah, USA) and Postview (Version 1.9.0, University of Utah, Musculoskeletal Research Laboratories and Columbia’s Musculoskeletal Biomechanics Laboratory, Columbia, Utah, USA). Tetrahedral meshes were generated from surface meshes via Preview (Figure 1C,D). The femur and tibia were designed with TRI3 elements (triangles), the collateral ligaments as TET4 (four-node tetrahedrons) and the cartilage covering the articular surfaces of the femur, tibia and the menisci with TET 10 (10-node tetrahedrons). In theory, hexahedral elements would be preferable for analysing pressure or shear forces by FEA [33], but they can hardly be generated automatically. In contrast, tetrahedrons can be generated automatically and 10-node tetrahedrons also produce satisfactory results [34]. A summary of all elements and formulas used during FEA is given in Supplementary Materials Table S1 and S1 Formulas.

### 2.4. Finite Element Model

Within the model, X was defined as mediolateral axis, Y as craniocaudal and Z as the vertical axis. Due to the complexity of the femoro–tibial joint, at this state only an abstracted model including bones, cartilaginous structures and ligaments without patellar or adjacent muscles was modelled considering only one degree of freedom in motion (along the craniocaudal axis (Y) of the joint).

The FEM “Open Knee–Generation 1” [35,36] was used as a reference for material properties. Femur and tibia were defined as rigid bodies following common practice in FEA [27,36]. For the articular cartilage surfaces of tibia and femur, a Mooney–Rivlin model was defined with the following material properties: density = 1.5 × 10^−9^ tons/mm^3^; Mooney–Rivlin material coefficient C_1_ = 0.856 MPa (Mega Pascal) Mooney–Rivlin material coefficient C_2_^X^ = 0 MPa; Bulk modulus (K+) = 8 MPa; X = C_2_ set to zero to get a Neo Hookean material [34,37].

For modelling the collateral ligaments, a nearly incompressible, transverse-isotropic hyper elastic fiber material on a Mooney–Rivlin basis was used (Table 1). The menisci were defined as a Fung orthotropic hyper elastic material (Table 2). Between the bone (non-deformable mesh) and cartilage surfaces (deformable mesh) as well as between the femur and the tibia (bone–bone contact) a rigid interface contact was employed. The anatomical center of rotation was set in a line through both origins of the collateral ligaments at the condyles of the femur [2]. Between the cartilage surfaces of femur/tibia and the menisci sliding contacts were defined, where the joint cartilage was chosen as “slave”, because of their smoother surface [37]. Friction was neglected in our model.

It was not possible to accurately reconstruct the geometry of the menisco–femoral and menisco–tibial ligaments from the MRI data, because of limited image resolution. Thus, the meniscal ligaments were simulated as “spring tied interface contacts” between surface nodes on the horns of the menisci and the tibia (Figure 1C,D). For the meniscal ligaments, linear stiffness values reported for bovine meniscal ligaments according to Villegas et al. (2007) were implemented [38].

As no data of equine tissue properties were available for the meniscal ligaments, bovine meniscal ligaments were implemented according to Villegas et al. [38]. For the caudal tibial ligament of the lateral meniscus and the cranial tibial ligament of the lateral meniscus an identical linear stiffness was supposed [39] (Lig. tibiale craniale menisci lateralis = 317 N/mm; Lig. tibiale craniale menisci medialis = 336 N/mm; Lig. tibiale caudale menisci lateralis = 317 N/mm; Lig. tibiale caudale menisci medialis = 381 N/mm). The contact area between the medial meniscus and the medial collateral ligament was defined as “Tied contact”. The meniscofemoral ligament was not simulated.

### 2.5. Boundary Conditions: Loads and Constraints

Four boundary conditions were defined for the FEA: (1) the degrees of freedom of the tibia were set zero; (2) the simulation range was set to 150° around the X-axis; (3) for simulating a ground reaction force of four N/kg bodyweight [39], a load of 384 N in the Z-direction was defined; (4) movement of the femur on the Y-axis and rotation around the Y-axis were set zero to simulate stabilizing tissue which was not part of the simulation. This set up (reduction of the motion to one degree of freedom) allowed the effects of stifle flexion without latero-medial rotation of the femur to be studied. Starting point was an angle of ca. 145° of the femorotibial joint reflecting a physiologic angle of an equine stifle joint while standing [1,2]. Although simulation of the full range of flexion (150°) was envisaged, only a range of motion of approximately 30° could be successfully simulated. Initial contact (force transmission to the menisci) started with the rotation.

The initial strain of the ligaments was set to zero based on the spiral configuration of the femoral condyles and the eccentric insertion of the collateral ligaments in relation to the axis of the joint. Movement tightens the ligaments and slows down joint motion when the joint moves toward the extended position [40]. Virtual flexion stopped at 117°.

### 2.6. Simulation

Load was applied in two steps: First, the Femur was loaded from zero to 384 N within 0.4 s. Then the rotation of the joint was simulated linearly form 145°–117° in 1.4 s.

By virtually marking ten points along the peripheral contour of both menisci not only the translocation of the whole menisci but also local deformation indicated by translocation of single distinct markers, could be analyzed

## 3. Results

The outcome of the FE-model simulation was restricted to the effects on the menisci.

At an angle of 117° the simulation stopped despite of multiple adjustments due to computational problems (“failure to converge”). However, computational simulation of approximately 30° flexion (ca. 145°–117°) with one degree of freedom proved to be feasible and predictive for pressure distributions, time and location of specific stress strain maxima as well as translocation and deformation of the equine menisci during gait.

### 3.1. Pressure Distribution

**Figure 2:**
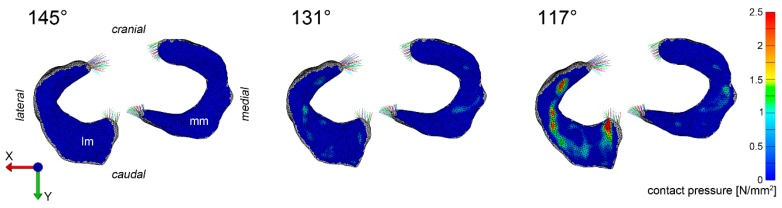
Pressure distribution maps depicting contact pressure on the medial and lateral meniscus. The contact pressure [N/mm^2^] is visualized for both menisci at 145°, 130° und 117°. Lm = lateral meniscus, mm = medial meniscus.

Pressure distribution varied between the menisci (medial and lateral) as well as within each individual meniscus. The simulation revealed an overall higher pressure load in the lateral meniscus compared to the medial with a distinct pressure peak of 4.75 N/mm^2^ in the caudal horn at a joint angle of 117°. Despite of this distinct pressure peak, loads were distributed evenly over the lateral meniscus ranging between 0.5 N/mm^2^ and 3.0 N/mm^2^ (Figure 2). For the medial meniscus no focal peak pressure as in the lateral meniscus was detected but an area of mildly pronounced pressure (1.5 N/mm^2^) at the attachment site of the medial collateral ligament at a joint angle of 117° (Figure 2).

### 3.2. Deformation and Displacement

**Figure 3:**
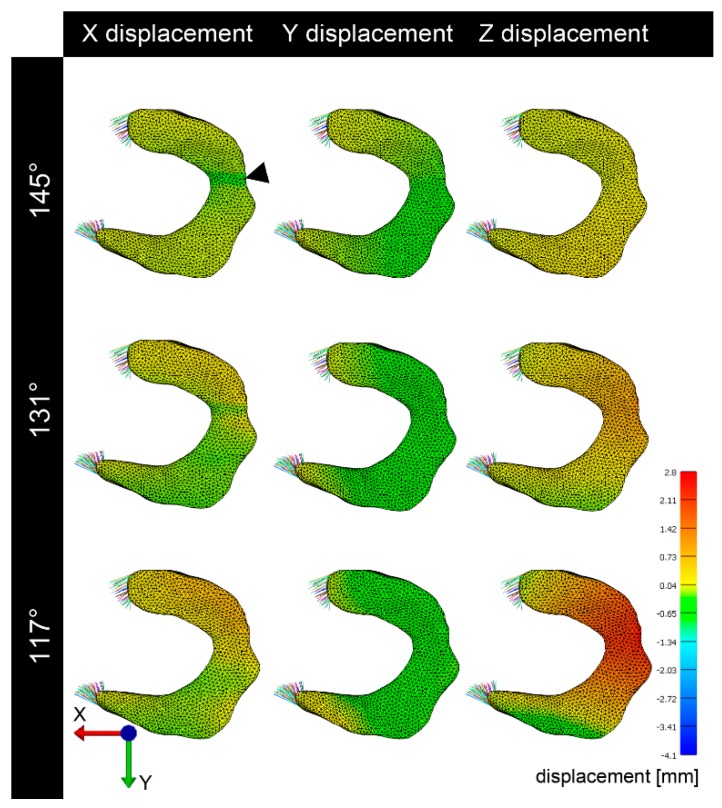
Translocation/deformation of the medial meniscus. Translocation of the medial meniscus in the X-axis is seen early in the simulation (145°) in particular in the attachment area of the medial collateral ligament (arrowhead). Strongest translocation in Y-axis is seen in the area of the meniscal ligament attachments. In the Z-axis, strongest translocation is seen at high flexion (117°) in the attachment area of the medial collateral ligament.

**Figure 4:**
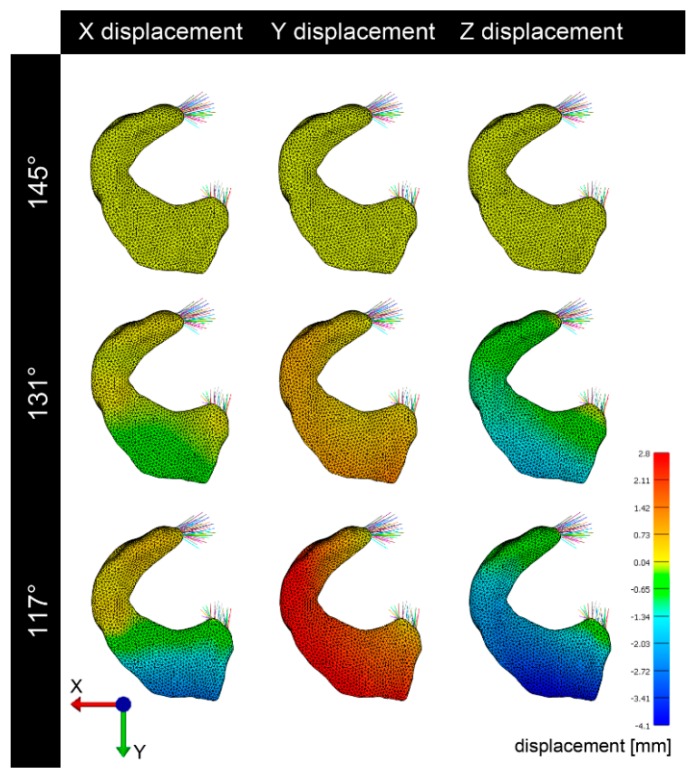
Translocation/deformation of the lateral meniscus. In the X-axis, the lateral meniscus shows some deformation. While the cranial horn shows little displacement, the caudal horn moves medially. Strong translocation of the lateral meniscus occurs in the Y-axis, showing that at high flexion (117°) the meniscus moves caudally. A remarkable translocation occurs also in the vertical (Z) axis, showing that at high flexion (117°) the meniscus is pushed distally.

**Figure 5:**
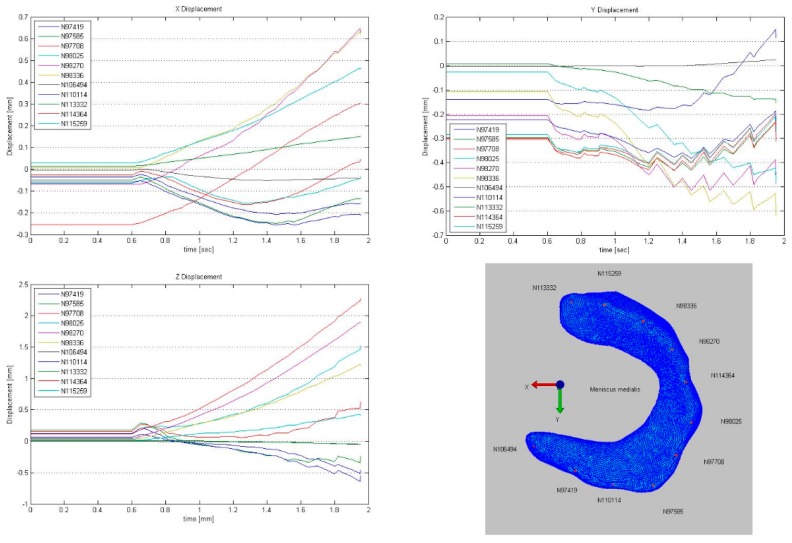
Displacement of ten virtually marked points at the peripheral contour of the medial meniscus during simulation.

**Figure 6:**
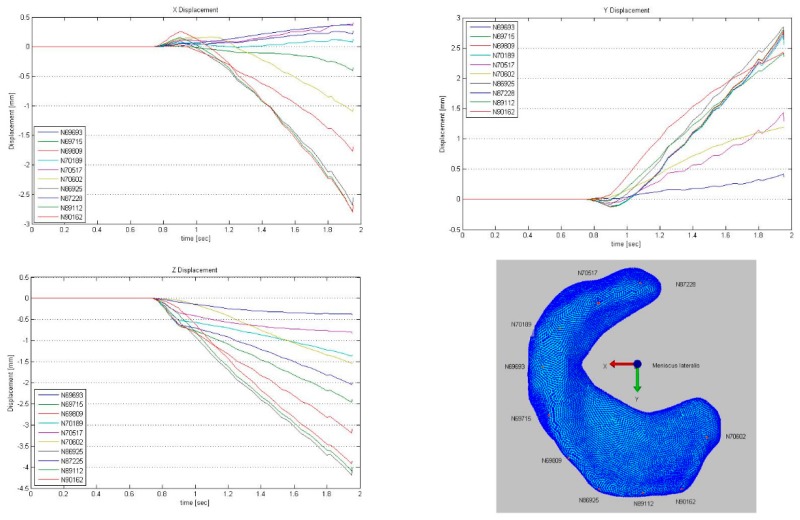
Displacement of ten virtually marked points at the peripheral contour of the lateral meniscus during simulation.

**Figure 7:**
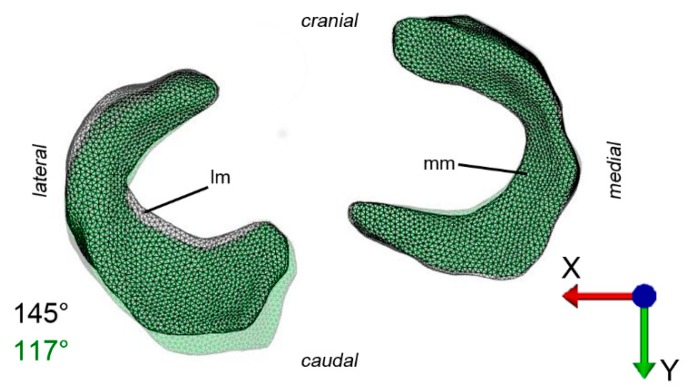
Visualization of maximum translocation/deformation of the medial and lateral meniscus. This semi-transparent overlay of 145°/117° in the X–Y plane shows that both translocation and deformation is higher in the lateral compared to the medial meniscus. Lm = lateral meniscus, mm = medial meniscus.

Overall, the medial meniscus was subjected to less deformation compared to the lateral (Figure 3, Figure 4, Figure 5, Figure 6 and Figure 7). Remarkable was an initial (load 384 N, joint angle 145°) slight deformation at the origin of the medial collateral ligament which remained consistent throughout the further simulation (Figure 3). The most pronounced deformation (load 384 N, joint angle 117°) of the medial meniscus in the direction of the X-axis was detected in the abaxial aspect of the cranial horn, in the direction of the Y-axis in the caudal horn and in Z direction at the attachment site of the medial collateral ligament (Figure 3).

The lateral meniscus was considerably more mobile and deformable than the medial (Figure 3, Figure 4, Figure 5, Figure 6 and Figure 7). For the lateral meniscus, in the X direction, the most pronounced deformation was detected in the caudal horn. In Y direction, a consistent craniocaudal translocation was observed in caudal direction mainly in the abaxial aspect of the meniscus with little to no variation from cranial to caudal. However, there was an increase in deformation from the axial to the abaxial aspect. Only the insertion areas of the cranial and caudal meniscotibial ligament were not subjected to deformation. In Z direction, meniscus displacement/deformation varied from the cranial to the caudal horn with a considerable distal translocation in the caudal horn and little displacement in the cranial horn (Figure 4).

### 3.3. Visualization of Pressure Distribution and Deformation/Translocation of the Menisci

To visualize pressure distribution on the menisci, contact pressure [N/mm^2^] was mapped on the menisci using a false-color lookup table at 145°, 131°, and 117°, respectively (Figure 2). Similarly, point translocations in X, Y, and Z were visualized with false colors again at 145°, 131°, and 117° for the medial (Figure 3) and lateral (Figure 4) meniscus. Ten points along the peripheral contour of the menisci were virtually marked and their change in position over the X-, Y- and Z-axis was evaluated at different joint angles to illustrate meniscus’ deformation and craniocaudal translocation during locomotion (Figure 5 and Figure 6). To show maximum deformation/translocation, a semi-transparent overlay image was generated from 145°/117° (Figure 7).

## 4. Discussion

FEA is a computational technique widely used in the field of comparative biomechanics [41] to study complex structures, their mechanical behavior and response to loading [42]. In the medical field FEMs including gait cycle load data may be used to illustrate dynamic joint performance and predisposition to injury of individual joint structures [43]. Another area of application could be the prediction of stress and strain changes following injury or (surgical) interventions (e.g., partial meniscectomy), or modelling the durability, resilience and shock absorption of implants/transplants [22]. Although a predictive FEM will not replace in vivo animal experimentation, it can potentially reduce the required number of animal trials.

We therefore aimed to develop a functional and predictive computational model of the equine stifle joint. However, the femoro–tibial joint is one of the most complex mammalian joints; hence modelling is equivalently multifaceted. Therefore a stepwise approach for the development of the FEM was chosen starting with a simplified model of the equine stifle (with one degree of freedom in motion, encompassing merely bones, cartilaginous structures and ligaments without patella or muscles). At later stages, further components will be added in the fashion of a modular add-on system.

The focus of the current paper was set on the menisci. As in humans [44] the equine menisci are multifarious tissues [13]. Similar to articular cartilage, known to show strain and time dependent property changes influenced by the contained collagen fibrils, fluid and proteoglycans [45], the menisci behave like viscoelastic, anisotropic, biphasic structures. The anisotropic and biphasic composition allows for optimal redirection of axial forces into radial strain and endurance of the compressive, tensile, shear and hoop stress [16]. Due to regionally differing proteoglycan (PG) and collagen contents, as well as collagen fiber orientation, the mechanical properties of the meniscus may vary greatly with location [46,47].

A clear limitation of the study is that friction of the joint was neglected. In combination with no initial stress on the ligaments this may lead to instability of the virtual model. This may be the reason why the lateral meniscus appears to be subjected to higher loads as compared to the medial. This is in contrast to previous studies and the clinical situation [1,48]. Usually it is the other way round. If there is the possibility to slip with a certain friction the relative position of the femur to the tibia can change (slide in place) before the maximum load is applied [40,48].

Although the presented FEM of the equine stifle joint at its current state allows only for the consideration of one degree of freedom (flexion of the femoro–tibial joint) and the range of flexion was limited to approximately 30° (145°–117°), it was possible to successfully simulate pressure distributions, identify time and location of specific stress strain maxima as well as translocation and deformation of the menisci during gait.

Geometric models obtained from MRI data were complemented 311 with data on tissue material properties available from studies on human and bovine femoro–tibial joint. The choice of material properties has considerable impact on the validity of the model [41]. Unfortunately equine specific data were not available. Therefore data obtained from comparable or anatomical similar structures from other species were implemented. Data from bovine meniscal ligaments [38] for example were considered similar because of the comparable equine and bovine anatomy and body size. Also human properties have been implemented due to the lack of data from comparable animal species. The human “Open knee(s) project” [35,36] served as general reference for designing the model. Human material properties can be assumed to be comparable. Not only does the equine stifle anatomy and meniscus histology [13] as well as the gait correspond to the human [12], but also the craniocaudal translocation of equine menisci during stifle flexion and extension is similar to that reported in humans [14]. In humans the highest radial displacement in response to loading (2 mm) was predicted for the lateral meniscus, however during the final stance phase the highest displacement was detected in the medial meniscus (2.5 mm) [43]. Fowlie et al. [1] described the translocation in equine menisci during gait and found a significantly bigger translocation of the lateral equine meniscus in craniocaudal direction compared to the medial. This and the fact that the cranial horn of the medial meniscus is the least mobile in the equine stifle joint [1] were confirmed by the present study, showing the lateral meniscus to be considerably more mobile and deformable than the medial (Figure 3, Figure 4, Figure 5, Figure 6 and Figure 7).

Pressure distribution varied between the menisci as well as within each individual meniscus. There was a much higher pressure load in the lateral meniscus with a distinct pressure peak in its caudal horn. Despite of this distinct peak, loads were distributed evenly over the proximal and central area of the lateral meniscus ranging between 0.5 N/mm^2^ and 3.0 N/mm^2^. This is interesting because the most commonly encountered meniscus injuries in horses are isolated lesions of the cranial horn of the medial meniscus and its meniscotibial ligament [15,17]. However, it is also known that these lesions typically result from overextension of the femoro–tibial joint during athletic use causing sudden compressive forces of 338 this region [15] which we were not yet able to model.

Ellenberger et al. [2] used anatomic landmarks of their flexion angle measurements which weren’t available for our model because of reduction in this model (Trochanter major cranialis–Tuberositas tibiae–Malleolus lateralis) and Fowlie et al. [1] were able to measure the angle directly with their experiment set up.

We used an earlier version of the model for the measurement in which parts of the diaphysis of the Femur and Tibia were still available. Two points along the cranial contour of the femur and tibia were marked and measured via four-point measurement. This allowed time dependent measuring of the stifle angle. Most likely, these angles are not entirely identical to Ellenberger et al. [2] or Fowlie et al. [1] but they a sufficient approximations.

For further enhancement of the presented meniscus FEM it will be necessary to refine some of the involved objects, especially the meniscal horn attachments and other femoro–tibial joint ligaments. This will require better delineation of the soft tissue components of the femoro–tibial joint, in particular the ligaments, in the images based on which the FEA model is created. However, recent studies have shown the importance of these attachments and how laxity of these structures may lead to loss of meniscal function [49]. Accurate modelling of the meniscal horn attachments is particularly important in the equine stifle because of the known involvement of the meniscotibial ligaments when it comes to meniscus injuries [15].

A computer tomographic contrast agent study would have probably increased the soft tissue identifiability and would have been preferable over the MRI images used in this study [50,51]. Nonetheless identifiability of the relevant structures in combination with the data implemented from other species was sufficient to allow realistic simulation of pressure distributions, time and location of specific stress strain maxima as well as translocation and deformation of the equine menisci. Furthermore, depth dependent fibril strains are considered to exhibit a fundamental role on tissue stresses [45]. Some human computational meniscus models have already incorporated collagen fibrils and anisotropic material properties to simulate the meniscus’ dynamic behavior [43,52,53,54] Hence, depth- and location specific histologic and mechanical properties will be adopted into the next generation of the FEA model based on data from our previous work [13,55]. Also the currently omitted stabilizing effect of ligaments and adjacent muscles should be included in future approaches.

## 5. Conclusions

In the current paper the FEA was used to explore the extent of meniscal extrusion and contact pressure distribution and to simulate meniscus’ translocation and deformation during locomotion. A functional FEM model can not only help identify segments in the stifle which are predisposed to injury, but also to better understand the progression of certain stifle disorders, simulate treatment/surgery effects and to optimize implant/transplant properties. To our knowledge this is the first prototype of an FEA model of the equine menisci simulating the effect of the joint angle to pressure distribution.

## Figures and Tables

**Table 1:** Collateral ligament material properties used for finite element analysis (FEA). Lig. Coll. Lat. = ligamentum collaterale laterale (lateral collateral ligament); Lig. Coll. Med. = ligamentum collaterale mediale (lateral collateral ligament) C_1_, C_2_: Mooney–Rivlin coefficients; K: Bulk modulus; C_3_: exponential stress coefficient; C_4_: Fiber uncrimping coefficient; C_5_: modulus of straightened fibers; Λm: fiber stretch of straightened fibers. C_2_^X^ = 0 set zero to get a neo Hookean material [34] density in tons/mm^3^, other units, if existing, in MPa (Mega Pascal).

Ligament	Density	C_1_	C_2_^X^	K	C_3_	C_4_	C_5_	Λ_m_
Lig. coll. Lat.	1.5 × 10^−9^	1.44	0	397	0.57	48	467.1	1.063
Lig. coll. Med.	1.5 × 10^−9^	1.44	0	397	0.57	48	467.1	1.063

**Table 2:** Meniscus material properties used for FEA. E_1,2,3_: Youngs Modulus E_1_, E_2_, E_3_; v_12_, v_23_, v_31_: Poisson’s ratio v_12_, v_23_, v_31_; G_12_, G_23_, G_31_: shear modulus G_12, 23, 31_; c: coefficient; K: bulk modulus. Density in tons/mm^3^, other units, if defined, in MPa (Mega Pascal). K and c are without effect for material behavior and served initial parameters.

Density	E_1_	E_2_	E_3_	v_12_	v_23_	v_31_	G_12_	G_23_	G_31_	c	K
1.5 × 10^−9^	125.0	27.5	27.5	0.1	0.33	0.1	2.0	12.5	2.5	1.0	10.0

## Supplementary Materials

The following are available online at https://www.mdpi.com/2076-2615/9/8/502/s1. Figure S1: Image processing and manual segmentation of the menisci. Summary of FE modelling parameters: Table S1: Summary of number of elements of all objects used in FEA, Formula S1: Material properties used for FEA.
