# Supplementary material for: Finite Element Modelling Simulated Meniscus Translocation and Deformation during Locomotion of the Equine Stifle
Source: Animals (Basel). 2019 Jul 31;9(8):502. doi: 10.3390/ani9080502 (PMC6720206; doi:10.3390/ani9080502)
Supplement: Supplementary file 1 [file animals-09-00502-s001.pdf]

Supplementary material: Figure S1

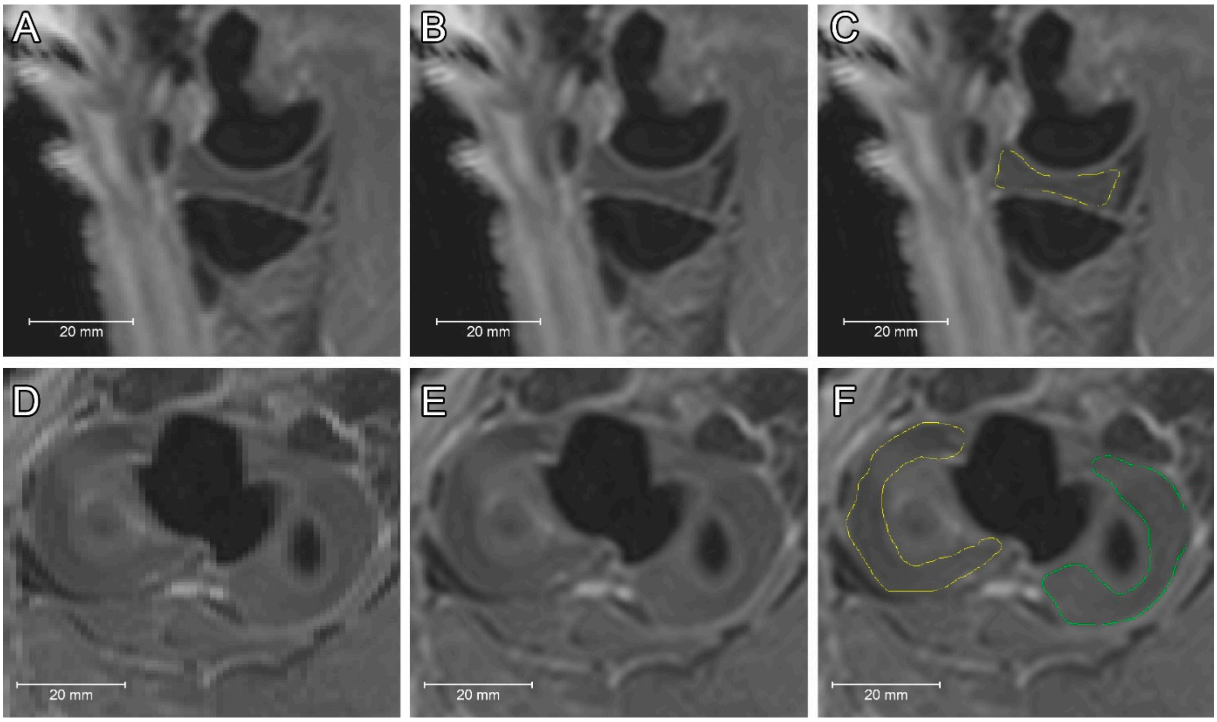

Figure S1. Image processing and manual segmentation of the menisci.

Supplementary material: Summary of FE modelling parameters

Table 1. Summary of number of elements of all objects used in FEA. Tri3: triangle elements; Tet4: 4-node tetrahedral elements; Tet10: 10-node tetrahedral elements.

**Object**	**Type**	**Number of Elements**
Femur	Tri3	25376
Tibia	Tri3	17690
Cartilage lat. Femurcondyle	Tet10	6615
Cartilage med. Femurcondyle	Tet10	8171
Cartilage lat. Tibia plateau	Tet10	4210
Cartilage med. Tibia plateau	Tet10	3463
Meniscus laterale	Tet10	15623
Meniscus mediale	Tet10	14732
Ligamentum collaterale laterale	Tet4	7078
Ligamentum collaterale mediale	Tet4	5922

S1 Formulas: Material properties used for FEA

We used the OpenKnee project as guidance and therefore the cartilage tissue on Femur and Tibia was defined as Mooney-Rivlin model (see formula).

$$\Psi = C_1\left(\tilde{I}_1 - 3\right) + C_2\left(\tilde{I}_2 - 3\right) + \frac{K}{2}(\ln J)^2 \qquad (1)$$

The Mooney-Rivlin model was also used by Erdemir und Sibole 2010 and Maas et al. 2016.

For further details on the used values see table below.

Density	C1	C2x	K+
1,5e-9	0,856	0	8

The approach chosen for the ligaments were similar (see Erdemir and Sibole 2010 and formulas and table below).

$$W = C_1\left(\tilde{I}_1 - 3\right) + C_2\left(\tilde{I}_2 - 3\right) + \frac{K}{2}(\ln(J))^2 + F(\tilde{\lambda}) \qquad (2)$$

Whereby

$$\tilde{\lambda}\frac{\delta F}{\delta \tilde{\lambda}} = \begin{cases} 0 & \tilde{\lambda} < 1 \\ C_3\left(e^{C_4\left(\tilde{\lambda}-1\right)} - 1\right) & 1 \le \tilde{\lambda} < \lambda_m \\ C_5 + C_6\tilde{\lambda} & \tilde{\lambda} \ge \lambda_m \end{cases} \qquad (3)$$

Ligament properties in table 1.

Menisci

Menisci were modelled as orthotropic hyper elastic material (formula below).

$$\Psi = \frac{1}{2}c\left(e^{\tilde{Q}} - 1\right) + U(J) \qquad (4)$$

Whereby

$$\tilde{Q} = c^{-1}\sum_{a=1}^{3}\left[2\mu_a M_a : \tilde{E}^2 + \sum_{a=1}^{3}\lambda_{ab}\left(M_a : \tilde{E}\right)\left(M_b : \tilde{E}\right)\right] \qquad (5)$$

Source: (Maas et al. 2016)
